# CORRELATES OF DEPRESSION/ANXIETY AND LITERACY IN FEMALE CAREGIVERS LIVING WITH HIV IN UGANDA AND MALAWI

**DOI:** 10.51894/001c.122811

**Published:** 2024-08-30

**Authors:** Tiffany Zheng, Jenus Shrestha, Itziah Familiar-Lopez

**Affiliations:** 1 College of Osteopathic Medicine Michigan State University; 2 Department of Psychiatry Michigan State University

## 8

### BACKGROUND

In sub-Saharan Africa, women face a multitude of stressors that can exacerbate mental health issues like depression and anxiety. We sought to evaluate how demographic factors are associated with depression and anxiety symptoms among women in Kampala, Uganda, and Blantyre, Malawi.

### METHODS

This is a secondary data analysis based on information from mothers of children participating in a randomized control trial of a cognitive gaming application for school-age children. Demographic data was collected through questionnaires and depression and anxiety symptoms were assessed with the Hopkins Symptom Checklist 25-item version. Descriptive statistics to summarize data and linear regression models were used to evaluate the association between literacy, educational level, and socioeconomic level with overall psychological distress, depression, and anxiety scores.

### RESULTS

The study conducted involved 149 participants in Uganda and 298 participants in Malawi of approximately 36 years of age. Most reported literacy (Uganda n = 136, 91.3%; Malawi n = 270, 90.6%) and secondary education as the highest level of education obtained (Uganda n = 77, 51.7%; Malawi n = 145, 48.7%). The mean anxiety score was 3.8 (Uganda) and 3.1 (Malawi), while mean depression score was 5.8 (Uganda) and 5.7 (Malawi). Age was not significantly associated with any of the outcomes. Higher depression symptoms were strongly and significantly associated with illiteracy in Uganda (mean = -4.80, p=0.01), and in Malawi (mean = -3.89, p=0.004). Lower educational levels showed a weaker association with depression symptoms (Uganda mean= -0.08, p=0.72; Malawi mean = -0.33, p=0.049). Higher distress scores were also significantly associated with no literacy (Uganda mean = -0.21, p=0.03; Malawi mean = -0.19, p=0.005), but not with educational level. In both Uganda and Malawi, anxiety symptoms were not significantly associated with literacy or educational level.

### CONCLUSIONS

Overall, literacy appears to be a protective factor for distress and depression symptoms in female caregivers living in Uganda and Malawi. This observed association suggests an important value to the cognitive processes of reading and writing that could reduce the risk of depression among this population.

